# Multi-omics insights into the response of the gut microbiota and metabolites to albendazole deworming in captive *Rhinopithecus brelichi*

**DOI:** 10.3389/fmicb.2025.1581483

**Published:** 2025-04-23

**Authors:** Xinxi Qin, Jincheng Han, Li Xi, Longfei Zhao, Zhiqiang Li, Yanyan Cui, Junfang Hao

**Affiliations:** ^1^College of Biology and Food, Shangqiu Normal University, Shangqiu, China; ^2^College of Veterinary Medicine, Northwest A&F University, Xianyang, China

**Keywords:** *R. brelichi*, gut microbiota, 16S rRNA gene, non-targeted metabolomics, correlations

## Abstract

**Background:**

Parasite infection and deworming treatment affect the host gut microbiota. Exploring the response mechanism of the gut microbiota in *Rhinopithecus brelichi* (*R. brelichi*) to albendazole deworming treatment is of great value for protecting this critically endangered species.

**Methods and results:**

This study used metataxonomics and metabolomics to explore the responses of the gut microbiota and metabolites of *R. brelichi* to albendazole deworming treatment. The results showed that deworming significantly reduced the eggs per gram of feces (EPG). The 16S rRNA gene sequencing results showed that the richness and diversity of the gut microbiota in *R. brelichi* after deworming were significantly increased. Meanwhile, deworming treatment also changed the composition of the gut microbiota. At the genus level, the *Christensenellaceae R7 group*, *UCG 002*, *UCG 005*, *uncultured rumen bacterium*, and *Rikenellaceae RC9 gut group* were significantly enriched in the pre-deworming samples. *Unclassified Muribaculaceae*, *Prevotella 9*, and *Bacteroides* were significantly enriched in the post-deworming samples. Metabolomics analysis revealed that the relative abundance of 382 out of 1,865 metabolites showed significant differences between the pre- and post-deworming samples. Among them, 103 metabolites were annotated based on the HMDB and mainly classified into Prenol lipids, Carboxylic acids and derivatives, and Organooxygen compounds, etc. The KEGG enrichment analysis result indicated that these metabolites were mainly involved in energy, amino acid, lipid, and purine metabolism. Correlation analysis showed that *Bacteroides* and *unclassified Muribaculaceae*, whose relative abundances were upregulated after deworming treatment, were positively correlated with Kaempferol, 5,7-Dihydroxy-3-methoxy-4′-prenyloxyflavone, Purpurin, and Rhein, which have anti-parasitic activities. The *Christensenellaceae R7 group*, with a downregulated relative abundance after deworming treatment, was not only negatively correlated with the above four metabolites, but also positively correlated with Retinyl beta-glucuronide, which is a storage form of vitamin A, and positively correlated with CDP-Choline, which increases the host’s susceptibility to *Entamoeba histolytica* and *Plasmodium falciparum*.

**Conclusion:**

This study emphasizes that deworming treatment has an impact on the gut microbiota and metabolic functions of *R. brelichi*. By exploiting the correlations between differential microbiota and metabolites, potential probiotics or prebiotics can be explored, thereby enhancing the efficiency of deworming and reducing its side effects.

## Introduction

*R. brelichi* is one of the snub-nosed monkeys belonging to the genus *Rhinopithecus* and is mainly distributed in the Fanjingshan National Nature Reserve in northeastern Guizhou, China (Yue et al., 2023). Due to the threats of habitat fragmentation, a long breeding cycle, and low genetic diversity, the remaining population of *R. brelichi* is only about 400 (Guo et al., 2020). Thus, it is listed at the critically endangered (CR) protection level in the IUCN Red List of Threatened Species^1^ (Guo et al., 2020). To protect this endangered *Rhinopithecus* species, artificial rearing strategies implemented in wildlife breeding centers and zoos have emerged as effective conservation measures. Snub-nosed monkeys possess a four-chambered stomach and a capacious digestive tract. However, this physiological structure renders their gastrointestinal microecology highly susceptible to perturbations from various factors, including diet, habitat, and parasites, etc. Regarding diet, the gut microbiota of wild *R. brelichi* continuously adjusted with the seasonal changes of food to maintain energy balance. For instance, Bacteroidota was significantly enriched in the spring group, while Actinomycetota was significantly enriched in the winter group (Guo et al., 2024). In terms of habitat, compared to the gut microbiota of wild *R. brelichi*, the ratio of Bacillota to Bacteroidota in captive *R. brelichi* was significantly decreased. Moreover, the relative abundances of bacterial communities associated with glycolysis, such as Prevotellaceae, Christensenellaceae, and Fibrobacteraceae, also changed significantly (Huang et al., 2024). Parasites are one of the pathogens threatening the health of snub-nosed monkeys. It has been reported that more than 40 species of ectoparasites and endoparasites have been found in snub-nosed monkeys, mainly including pinworms, roundworms, whipworms, and protozoa, etc (Wang et al., 2019; Hasegawa et al., 2020). Studies have shown that gut bacterial communities and parasites have complex interactions (Nemathaga et al., 2023). *Strongylid nematodes* showed significant covariations with Prevotellaceae and Rikenellaceae in the gut of gorillas (Mason et al., 2022). Montero et al. (2021) found that helminth infection reduced the difference in gut microbiota among individual mouse lemurs (Montero et al., 2021). In colobus monkeys and baboons, intestinal *Strongyloides* and *Trichuris* infections were negatively and positively correlated with gut microbiota richness, respectively. In colobus monkeys infected with *Strongyloides*, representative bacterial taxa such as Ruminococcaceae, Lachnospiraceae, and *Pseudoflavonifractor* were significantly enriched (Barelli et al., 2021). Studies have also shown that parasitic infection can cause changes in the intestinal metabolomics of the host. *Toxocara canis* infection led to a significant decrease in intestinal metabolites involved in the tricarboxylic acid cycle, bile secretion, and glycolysis pathways (Wang N. et al., 2024).

Parasite investigation and deworming are effective ways to reduce the incidence of parasitic diseases and parasite-related enteric dysbacteriosis in captive snub-nosed monkeys. Albendazole is an imidazole derivative with broad-spectrum anthelmintic effect. It can be clinically used to expel pinworms, roundworms, whipworms, tapeworms, hookworms, and *Strongyloides*, etc (Chai et al., 2021). When albendazole is metabolized to sulfoxide or sulfones in animals, it can inhibit the uptake of glucose and cause glycogen depletion in the parasite, or inhibit the parasite fumarate reductase system to hinder the production of ATP. Ultimately, this makes the parasite unable to survive (Ignacio et al., 2023). However, the potential antimicrobial properties of albendazole and the elimination of parasites may lead to changes in the gut microbiota. It has been found that patients treated with albendazole had significantly reduced helminths carrier rates and gut microbiota richness. The relative abundance of Clostridiales significantly increased, while that of Enterobacteriaceae significantly decreased (Easton et al., 2019). Appiah-Twum et al. (2023) found that hookworm infection increased the richness of human gut microbiota. Moreover, the relative abundance of Clostridia may be up-regulated through the pyruvate ferredoxin oxidoreductase pathways. The richness and composition of the gut microbiota in the infected group changed significantly before and 10–14 days after treatment with 400 mg albendazole, and the composition of the gut microbiota in the successfully dewormed patients tended to be similar to that in the uninfected group (Appiah-Twum et al., 2023). Similarly, Tee et al. (2022) discovered that infections by helminths and *Trichuris* enhanced the richness of the human gut microbiota. They also found a significant positive correlation between the quantity of infection and the richness of the microbiota. After subjects received 400 mg of albendazole orally for three consecutive days, a total of 93 bacterial taxa exhibited significant alterations in their relative abundances on the 21st day. Albendazole also affected the metabolic process of bacterial communities by up-regulating the L-glutamate degradation V pathway and down-regulating phosphoenolpyruvate carboxylase gene expression (Tee et al., 2022).

The present study was designed to explore the effects of albendazole deworming treatment on the gut microbiota and metabolites of snub-nosed monkeys. The goal was to furnish data for the scientific development of deworming regimens and feeding management strategies for endangered captive animals. Through comparative analysis of the differences in the gut microbiota and metabolic functions of *R. brelichi* before and after albendazole deworming, biomarkers with significant alterations were identified, and the matters needing attention during albendazole deworming were discussed.

## Materials and methods

### Sample collection

Ten *R. brelichi* (aged 2–8 years) from Beijing Zoo received routine albendazole deworming in November 2023. In the three-month period prior to deworming and following the treatment process, these monkeys maintained a normal mental state, exhibited no signs of diarrhea, and did not receive any medications other than albendazole. Their diet consisted of fresh leaves, vegetables, fruits, cooked cornbread, and eggs. The breeder mixed albendazole (200 mg/tablet, Smithkline Pharmaceutical Co., LTD.) into the food and administered it to each monkey at a dosage of 10 mg/(kg⋅bw), once a day for three consecutive days. Fresh fecal samples were collected both prior to deworming and 7 days after the deworming procedure.

### Anthelminthic effect of albendazole

Fresh feces of 1.0 g were taken from each sample. The eggs of parasites were floated using the saturated salt flotation method (Adhikari et al., 2023). Then, the eggs were counted by Mc-Master’s method (Lejeune et al., 2023) under an optical microscope (Olympus BX51 Microimaging System, Japan) to calculate EPG.

### High throughput sequencing of the V3–V4 region of 16S rRNA gene in gut microbiota

A 0.5 g fecal sample was weighed. The E.Z.N.A™ Mag-Bind Soil DNA Kit (Omega Bio-Tek, USA) was used to extract the total bacterial DNA, and 1% gel electrophoresis was used to detect it. Then, the DNA concentration was quantitatively determined by Qubit^®^ 4.0 (ThermoFisher Scientific, USA) after processing the DNA sample with the Qubit 1 × dsDNA HS Assay Kit. Universal primers (341F: CCTACGGGNGGCWGCAG; 805R: GACTACHVGGGTATCTAATCC) was used for PCR amplification of the 16S rRNA gene V3-V4 region (Herlemann et al., 2011). Amplification reaction system: 2 × Hieff^®^ Robust PCR Master Mix (Yeasen, China) 15 μL, Primer F 1 μL, Primer R 1 μL, DNA 10 ng, ddH_2_O supplemented to 30 μL. PCR reaction conditions: 94°C for 3 min; 5 cycles (94°C 30 s, 45°C 20 s, 65°C 30 s); 20 cycles (94°C 20 s, 55°C 20 s, 72°C 30 s); 72°C for 5 min. The quality and concentration of the products were detected by 2% gel electrophoresis and Qubit^®^ 4.0 fluorescence quantifier. Libraries were constructed using the TruSeq^®^ Nano DNA Kit (Illumina, USA). Based on the Illumina NovaSeq 6000 platform, paired-end (2 × 250 bp) was used for high-throughput sequencing of the constructed libraries.

### Bioinformatics analysis based on sequencing data

Adapters, primers, and low-quality sequences were removed from the raw reads using Trimmomatic v0.33 and cutadapt 1.9.1 software. In QIIME2 2020.6 software, the DADA2 plug-in was used for denoising, double-ended sequence splicing, and removal of chimeric sequences. The processed reads were clustered into Amplicon sequence variants (ASVs) with 99% sequence identity. The Silva 138 database (Wang, 2023) was used as a reference to annotate the ASVs. The microbiota diversity and composition were evaluated using the q2-diversity plug-in in QIIME2 2020.6 software. The Wilcoxon rank-sum test was employed to analyze the significance of differences between groups, and the *P*-values were corrected by FDR. The Linear discriminant analysis effect size (LEfSe) software was utilized to search for biomarkers with statistical differences between groups based on LDA ≥ 4 and *P* < 0.05. Spearman rank correlation analysis was used to construct an correlation network diagram among bacterial communities.

### Metabolites extraction

Added 1 mL of cold extraction solution (methanol: acetonitrile: water = 2:2:1, v/v) to 50 mg fecal sample and vortexed it for 30 s. Ultrasonic treatment was performed at 4°C, 45 Hz for 10 min. Then, the sample was placed at −20°C for 30 min, followed by centrifuged at 4°C, 12,000 rpm for 15 min. The supernatant containing metabolites was taken out, and 500 μL of it was placed in an EP tube and dried in a vacuum concentrator. After adding 160 μL of extraction solution (acetonitrile: water = 1:1, v/v) to the dried metabolites, vortexed the mixture for 30 s and then sonicated it in an ice bath for 10 min. The sample was centrifuged at 4°C, 12,000 rpm for 15 min, and then 120 μL of the supernatant was taken and placed in an UHPLC sample vial. quality control (QC) samples were formed by mixing 10 μL of supernatant from each sample, and a QC sample was inserted after every 5 samples in the analysis process to investigate the repeatability of the entire analysis process.

### UHPLC-Q-TOF-MS/MS analysis

Non-target metabolomics analysis of intestinal metabolites was performed by ultra-high performance liquid chromatography coupled with quadrupole tandem time-of-flight mass spectrometry (UHPLC-Q-TOF-MS/MS) in positive and negative ion mode. Chromatographic conditions: Acquity UPLC HSS T3 column (1.8 μm, 2.1 × 100 mm, Waters Corporation, USA). Mobile phase A: 0.1% formic acid, Mobile phase B: 0.1% acetonitrile formate solution. The injection volume was 1 μL and the flow rate was 400 μL/min. The elution gradients were 2% B (0–0.25 min), 2%–98% B (0.25–10 min), 98% B (10–13 min), 98%–2% B (13–13.1 min), 2% B (13.1–15 min). Ion source: electrospray ionization interface (ESI). Capillary voltage: 2,500 V (positive ion mode) or −2,000 V (negative ion mode). Cone voltage: 30 V. Ion source temperature: 100°C. Collision energy (CE): 20–60 V. Mass-to-charge ratio (m/z) collection range: 50–1,200. MassLynx V4.2 software (Waters, USA) was used for primary and secondary mass spectrometry data acquisition.

### Metabolomics analysis

Progenesis QI 2.3 (Waters, USA) was used to process the data obtained from UHPLC-Q-TOF-MS to acquire information such as retention time, mass/charge ratio, and peak intensity, etc. Orthogonal projections to latent structures-discriminant analysis (OPLS-DA) was used to observe the variability of metabolite differences between groups and between samples within groups, and the stability of the OPLS-DA model was evaluated using 7 cycles of cross-validation. Variable importance in Projection (VIP) was calculated based on the OPLS-DA model, and the Fold change (FC) was calculated according to the quantitative data of metabolites. VIP > 1, FC > 1.2 or FC < 0.83, and *P* < 0.05 were used as the criteria to screen out the differential metabolites between pre- and post-deworming samples. Volcanic mapping of differential metabolites was performed based on FC and *P*-values. The pheatmap and RColorBrewer packages in R 3.1.1 software were used to collect and combine the differential metabolites identified by all comparison combinations, and the clustering heatmap of this set in all samples was drawn. The metabolite information was obtained by comparing MS and MS/MS spectrometry with the Human Metabolome database (HMDB) (Wishart et al., 2022). The Receiver operating characteristic (ROC) curves were plotted for the differential metabolites, and the area under the curve (AUC) was calculated. KEGG pathway enrichment analysis was performed for differential metabolites by Over representation analysis (ORA) (Kanehisa et al., 2025). Relationships between variables were assessed with Spearman rank correlation analysis.

## Results

### Anthelminthic effect of albendazole

**FIGURE 1:**
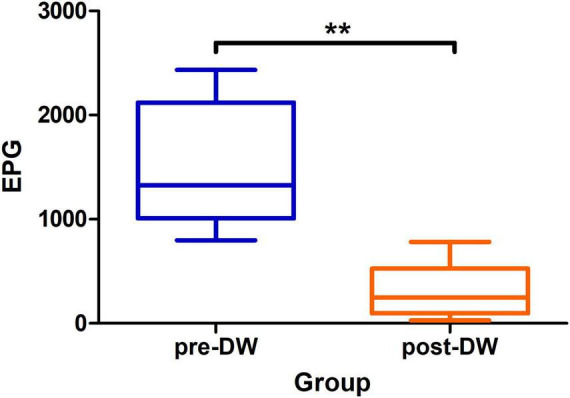
Eggs per gram of feces before and after deworming treatment with albendazole. pre-DW: fecal samples before deworming treatment; post-DW: fecal samples after deworming treatment. Paired *t*-test: **P < 0.01.

Compared with the number of 1,472.6 ± 605.6 EPG in the feces before deworming, the number of EPG in the feces significantly decreased to 296.1 ± 255.2 after deworming (*P* < 0.01) (Figure 1).

### Effect of deworming treatment on the diversity of gut microbiota

**FIGURE 2:**
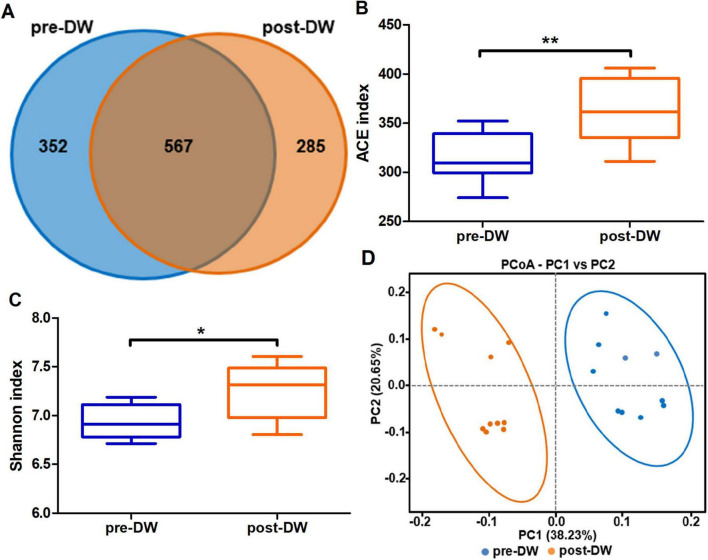
Effect of deworming treatment on microbiota diversity in the gut of *R. brelichi*. **(A)** Shared and unique ASVs between the pre- and post-deworming groups were visualized by Venn diagram. **(B)** ACE index. **(C)** Shannon index. **(D)** PCoA plot of beta diversity of gut microbiota before and after deworming. pre-DW: fecal samples before deworming treatment; post-DW: fecal samples after deworming treatment. Wilcoxon rank sum test, false discovery rate (FDR) correction: *P < 0.05, **P < 0.01.

A total of 1,604,220 pairs of reads were obtained from 20 fecal samples, after quality control and splicing of double-ended reads, 1,599,647 pairs of clean reads were generated, and 1,376,531 pairs of non-chimeric reads were obtained by DADA2 denoising (Supplementary Table 1). Among the 1,204 clustered ASVs, 567 ASVs were shared by both groups (Figure 2A). The ACE index and Shannon index were used to evaluate the alpha diversity of gut microbiota before and after deworming. The results showed that the ACE index (*P* < 0.01) and Shannon index (*P* < 0.05) of gut microbiota after deworming were significantly higher than those before deworming (Figures 2B, C). The PCoA analysis showed significant differences in the composition of gut microbiota before and after deworming (Figure 2D). The differences in beta diversity (PERMANOVA: R^2^ = 0.357, *P* = 0.001; ANOSIM: *R* = 0.847, *P* = 0.001) indicated that deworming treatment was closely related to the difference in microbiota composition between groups (Supplementary Figure 1).

### Variations of gut microbiota with deworming treatment

**FIGURE 3:**
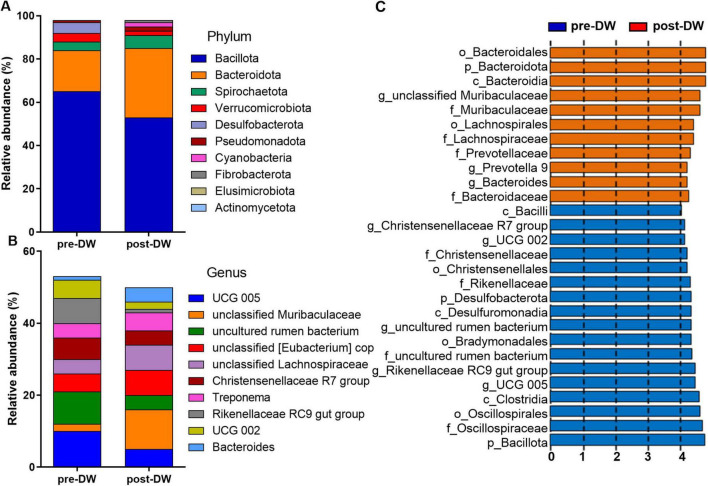
Effect of deworming treatment on gut microbiota composition of *R. brelichi*. **(A)** Species distribution of the top ten phyla pre- and post-deworming treatment. **(B)** Species distribution of the top ten genera pre- and post-deworming treatment. **(C)** LEfSe histogram showing differential biomarkers of gut microbiota in pre- and post-deworming groups. pre-DW: fecal samples before deworming treatment; post-DW: fecal samples after deworming treatment.

The histogram of species distribution showed that the predominant phyla in both groups were Bacillota and Bacteroidota (Figure 3A). The relative abundances of 7 phyla (58.33%) changed significantly before and after deworming (*P* < 0.01), including Bacillota, Bacteroidota, Pseudomonadota, Cyanobacteria, Fibrobacterota, Desulfobacterota, and Campylobacterota (Supplementary Table 2). The predominant genus in the microbiota before deworming was the *UCG 005* (10.91%) from Oscillospiraceae, while the predominant genus after deworming was the unclassified genus (11.37%) from the family Muribaculaceae (Figure 3B). The relative abundances of 26 (17.33%) genera changed significantly before and after deworming (Supplementary Table 3). The results of LEfSe showed that before deworming, the gut of *R. brelichi* was enriched with bacterial communities from Bacillota (Christensenellaceae, *Christensenellaceae R7 group*, Oscillospiraceae, *UCG 002, UCG 005*, and uncultured rumen bacterium from Bradymonadales) and Bacteroidota (Rikenellaceae and *Rikenellaceae RC9 gut group*). After deworming, the gut of *R. brelichi* was mainly enriched with bacterial communities from Bacteroidota, namely Bacteroidaceae, *Bacteroides*, Muribaculaceae, *unclassified Muribaculaceae*, Prevotellaceae, and *Prevotella 9*. In contrast, only Lachnospiraceae from Bacillota was enriched in the gut microbiota after deworming (Figure 3C).

### Correlation network analysis of the gut microbiota

**FIGURE 4:**
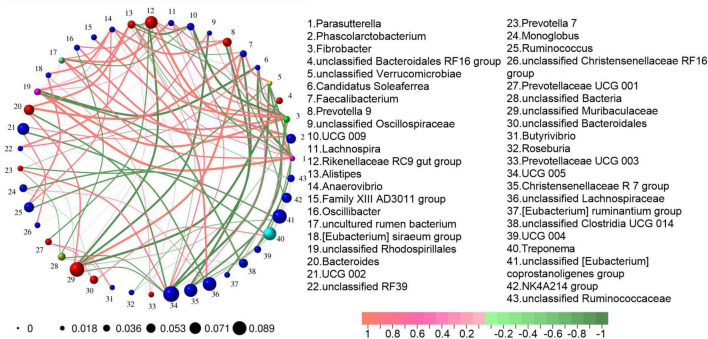
Correlation network analysis of gut microbiota. Construction criteria: The absolute value of the correlation coefficient is greater than 0.1 and the *P*-value is less than 0.05. The color of the connecting line represents the correlation between two nodes. Red indicates a positive correlation, while green indicates a negative correlation. The thickness of the connecting line represents the weight value derived from the correlation analysis. The thicker the line, the larger the weight value. The numbers outside the nodes indicate the bacterial genus represented by each node. The size of the nodes represents the average abundance of the bacterial genus.

A Spearman rank correlation analysis was conducted on the genus level microbiota. The analysis was based on the criteria of an absolute correlation value greater than 0.1 and a *P*-value less than 0.05. The results are presented in Figure 4. The *Christensenellaceae R7 group* exhibited an extremely strong negative correlation with *Prevotella 9* (−0.8114) and a strong positive correlation with *UCG 002* (0.6526). *UCG 005* showed an extremely strong negative correlation with *Faecalibacterium* (−0.8105) and unclassified *Verrucomicrobia* (−0.8188), a strong negative correlation with *Bacteroides* (−0.7489), and a strong positive correlation with the *Rikenellaceae RC9 gut group* (0.7218). The uncultured rumen bacterium had an extremely strong positive correlation with *Lachnospira* (0.8015), and strong positive correlations with *Bacteroides* (0.6318) and *Prevotella 9* (0.6220). The *Rikenellaceae RC9 gut group* demonstrated an extremely strong positive correlation with *Phascolarctobacterium* (0.8617). *Bacteroides* had an extremely strong positive correlation with *Faecalibacterium* (0.8150). *Unclassified Muribaculaceae* demonstrated an extremely strong positive correlation with *Faecalibacterium* (0.8150), a strong positive correlation with *Bacteroides* (0.7308), and extremely strong negative correlations with the *Rikenellaceae RC9 gut group* (−0.8000), unclassified *Oscillospirales* (−0.8045), and unclassified *Bacteroidales RF16 group* (−0.8189), and a strong negative correlation with *UCG 005* (−0.7068).

### Variations of fecal metabolic phenotypes with deworming treatment

**FIGURE 5:**
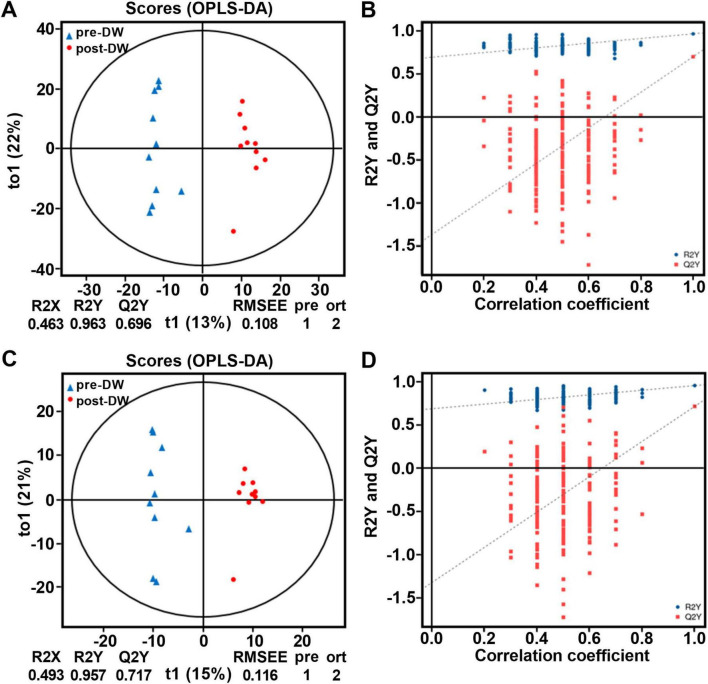
OPLS-DA analysis and permutation test of OPLS-DA of fecal metabolites between pre- and post-deworming groups. **(A)** OPLS-DA score plot in positive ion mode. **(B)** Permutation test of the OPLS-DA model in positive ion mode. **(C)** OPLS-DA score plot in negative ion mode. **(D)** Permutation test of the OPLS-DA model in negative ion mode. pre-DW: fecal samples before deworming treatment; post-DW: fecal samples after deworming treatment.

A total of 1,865 metabolites were identified from the 20 fecal samples (positive mode: 1,226, negative mode: 639) and annotated based on HMDB 4.0 database. At the superclass level, the metabolites in fecal samples were mainly composed of Organoheterocyclic compounds (35.18%), Lipids and lipid-like molecules (26.98%), Organic acids and derivatives (13.36%), Benzenoids (12.80%), and Phenylpropanoids and polyketides (6.49%) (Supplementary Table 4). OPLS-DA results showed that the metabolic phenotypes in the gut of *R. brelichi* were significantly different between the pre- and post-deworming groups (Figures 5A, C). In the OPLS-DA scoring chart, Q^2^Y values were 0.696 and 0.717 in positive and negative modes, respectively, indicating that the model has good stability and reliability. The positive slope of Q^2^Y fitting regression line in the permutation test of OPLS-DA model indicated that the OPLS-DA model was meaningful, and the blue dots are generally located above the red dots, indicating that the training set and test set had good independence (Figures 5B, D).

### The difference of metabolic profiles before and after deworming

**FIGURE 6:**
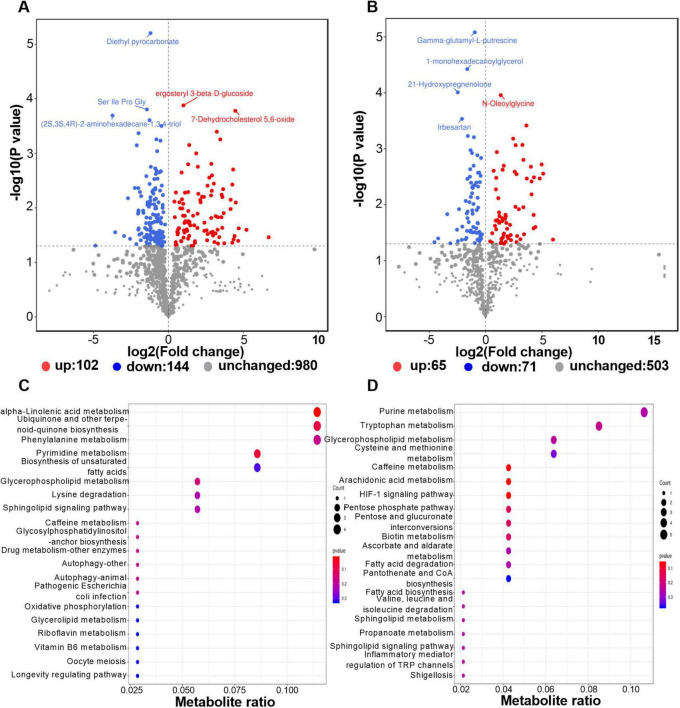
Distribution of the differential metabolites and enrichment analysis of their involved metabolic pathways. **(A)** Volcano plot of differential metabolites in positive ion mode. **(B)** Volcano plot of differential metabolites in negative ion mode. **(C)** KEGG pathway enrichment dotplot in postive ion mode. **(D)** KEGG pathway enrichment dotplot in negative ion mode.

The volcano plot of differential metabolites showed that after deworming treatment, 167 metabolites were significantly upregulated and 215 were significantly downregulated in the gut of *R. brelichi* (Figures 6A, B). These 382 differential metabolites were annotated to 103 taxonomies in the HMDB database. When ranked according to the number of metabolites they contained, the top 10 taxonomies were Prenol lipids (14.56%), Carboxylic acids and derivatives (12.62%), Organooxygen compounds (12.62%), Benzene and substituted derivatives (6.80%), Glycerophospholipids (6.80%), Fatty Acyls (5.83%), Steroids and steroid derivatives (4.85%), Phenols (3.88%), Glycerolipids (2.91%), and Organonitrogen compounds (2.91%). Among them, the relative abundances of metabolites belonging to Prenol lipids, Fatty Acyls, and Organonitrogen compounds were generally downregulated, while those belonging to Glycerophospholipids were generally upregulated (Supplementary Table 5). These metabolites with significant changes were mainly enriched in KEGG pathways such as ABC transporters, Phenylalanine metabolism, Purine metabolism, Ubiquinone and other terpenoid-quinone biosynthesis, Glycerophospholipid metabolism, Pyrimidine metabolism, Tryptophan metabolism, alpha-Linolenic acid metabolism, Bile secretion, Biosynthesis of amino acids, and Biosynthesis of unsaturated fatty acids (Figures 6C, D).

**FIGURE 7:**
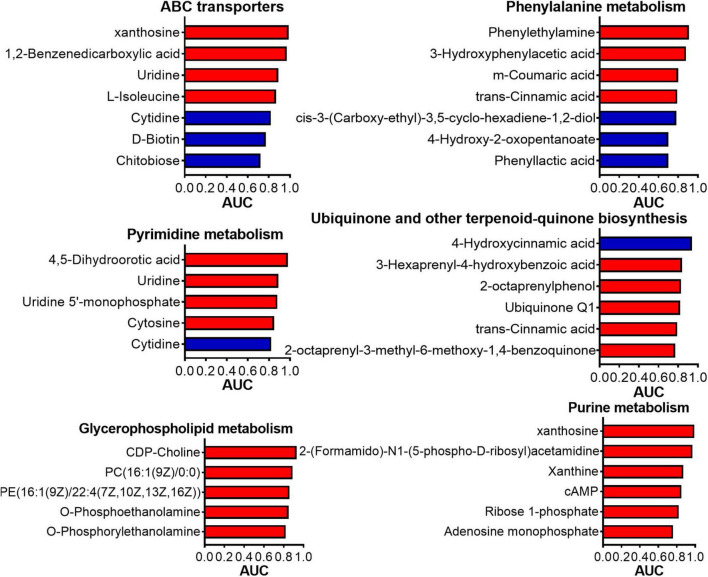
The AUC of each metabolite in the KEGG pathways that enriched the most differential metabolites. AUC: Area Under Curve.

The results of the ROC analysis indicated that, among the identified differential metabolites, 96 metabolites exhibited an AUC exceeding 0.9 (Supplementary Table 6). This finding suggested a significant correlation between the alterations in these metabolites and the deworming treatment. Notably, 24 out of the 96 metabolites were successfully annotated to metabolic pathways within the KEGG database. Among these 24 metabolites, 5 were downregulated, while the remaining 19 were upregulated (Supplementary Table 7). Figure 7 depicts the AUC values of the metabolites in the top 6 most enriched KEGG pathways for the differential metabolites. Evidently, xanthosine, which participated in both the ABC transporters pathway and Purine metabolism, demonstrated an impressively high AUC of 0.99 (log2 Fold Change, log2FC: 3.36). 1,2-Benzenedicarboxylic acid, being involved in the ABC transporters pathway, had an AUC of 0.97 (log2FC: 4.32). Phenylethylamine, associated with the Phenylalanine metabolism, showed an AUC of 0.91 (log2FC: 3.31). 2-(Formamido)-N1-(5-phospho-D-ribosyl) acetamidine, which was engaged in Purine metabolism, had an AUC of 0.97 (log2FC: 4.27). 4-Hydroxycinnamic acid in the Ubiquinone and other terpenoid-quinone biosynthesis had an AUC of 0.94 (log2FC: −1.03). CDP-Choline, which was involved in the Glycerophospholipid metabolism, had an AUC of 0.93 (log2FC: 2.27). 4,5-Dihydroorotic acid, associated with the Pyrimidine metabolism, had an AUC of 0.98 (log2FC: 4.98). These metabolites, which were not only annotated to metabolic pathways but also boasted high AUC values, are highly likely to be the key biomarkers in this study.

### Correlation analysis between differential gut microbiota and metabolites

**FIGURE 8:**
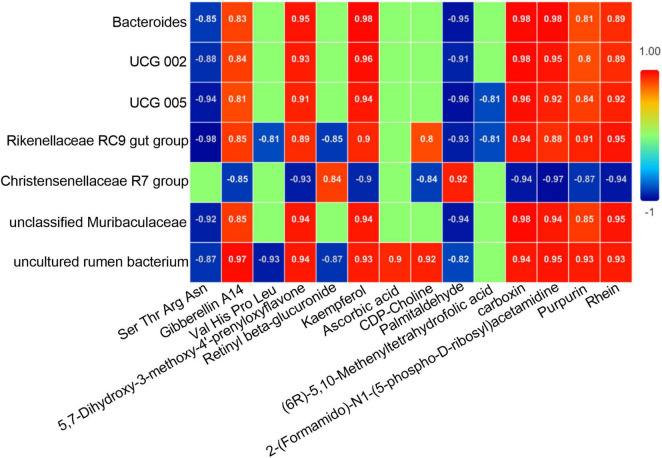
Correlation heatmap of the differential microbiota and differential metabolites with an AUC over 0.9. Correlations are indicated by colors and numbers. Among the colors, red represents positive correlation, blue represents negative correlation, and green represents no correlation. The number in the color block represents the degree of correlation. The closer the absolute value is to 1, the higher the correlation between the two factors; the closer the absolute value is to 0, the lower the correlation between the two factors.

Spearman correlation analysis was conducted between the differential microbiota (bacterial genera with LDA ≥ 4 and *P* < 0.05) and differential metabolites with an AUC over 0.9 based on deworming treatment. As shown in Figure 8, there were close relationships between the differential microbiota and metabolites. There were not only numerous correlations between the same bacterial community and diverse metabolites, but also varying degrees of correlations between the same metabolite and different bacterial communities. After deworming treatment, the *unclassified Muribaculaceae* and *Bacteroides* that were significantly enriched in the gut of *R. brelichi* showed a similar trend of correlation with the differential metabolites, presenting a significant negative correlation with Ser Thr Arg Asn and Palmitaldehyde, and a significant positive correlation with Gibberellin A14, 5,7-Dihydroxy-3-methoxy-4′-prenyloxyflavone, Kaempferol, carboxin, 2-(Formamido)-N1-(5-phospho-D-ribosyl) acetamidine, Purpurin, and Rhein. Notably, some bacterial communities with significantly reduced relative abundances after deworming treatment, such as *UCG 002*, *UCG 005*, *Rikenellaceae RC9 gut group* and *uncultured rumen bacterium*, showed similar correlations with the above 9 metabolites as *unclassified Muribaculaceae* and *Bacteroides*, indicating that the changes in the abundance of these metabolites are not only due to the increased abundance of certain communities, but also to the reduction of the above 4 communities in the gut, which relatively promotes the production of these metabolites. *Christensenellaceae R7 group* was generally negatively correlated with the differential metabolites with an AUC over 0.9. It was negatively correlated with Gibberellin A14, 5,7-Dihydroxy-3-methoxy-4′-prenyloxyflavone, Kaempferol, CDP-Choline, carboxin, 2-(Formamido)-N1-(5-phospho-D-ribosyl) acetamidine, Purpurin, and Rhein, and positively correlated only with Retinyl beta-glucuronide and Palmitaldehyde.

## Discussion

In this study, multi-omics approaches were used to analyze the changing characteristics of the gut microbiota and metabolites in *R. brelichi* before and after deworming treatment, with the aim of exploring potential biomarkers. Captive *R. brelichi* were administered albendazole at a dosage of 10 mg/(kg⋅bw) for three consecutive days for deworming. The deworming regimen adopted in this research led to a significant 79.89% decrease in the EPG levels of *R. brelichi*. Previous research has shown that *R. roxellana* were administered albendazole at a dosage of 10 mg/(kg⋅bw), with two treatments within a three-month period. The EPG reduction rate was 7.8%–73.2% for the first treatment and 52.6%–52.8% for the second treatment (Ren et al., 2025). Compared to this previous report, the deworming protocol used in the present study exhibited a relatively more effective deworming outcome. Based on the deworming spectrum of albendazole, it is hypothesized that the reduction in EPG is mainly attributed to the decline in the carriage rate of parasites such as nematodes, cysticerci, and echinococci (Chai et al., 2021).

Based on metataxonomic analysis, we observed significant changes in the diversity and composition of the gut microbiota after deworming treatment. In the literatures, the responses of gut microbiota diversities to deworming vary, which is closely associated with the deworming approach, type of parasitic infection, and metabolic shifts (Easton et al., 2019; Peachey et al., 2019; Tee et al., 2022; Appiah-Twum et al., 2023). In this study, compared with the pre-deworming samples, on the 7th day after deworming treatment, both the richness and evenness of the gut microbiota increased significantly. It is speculated that this is related to the alleviation of the parasite infection status. After deworming treatment, the gut microbiota of *R. brelichi* was enriched with bacterial communities from two orders, namely Lachnospirales (including Lachnospiraceae) and Bacteroidales (including Bacteroidaceae, *Bacteroides*, *Muribaculaceae*, *unclassified Muribaculaceae*, Prevotellaceae, and *Prevotella 9*). Lachnospiraceae, *Bacteroides*, and *Prevotella 9* are important bacterial communities, which not only play a crucial role in carbohydrate utilization, especially in the decomposition of complex plant fibers, but also have functions such as regulating lipid metabolism, maintaining the intestinal barrier, and regulating the microbial balance (Alqahtani et al., 2024; Liu et al., 2024; Wang D. et al., 2024; Yosi et al., 2024). Meanwhile, we also found that the deworming treatment led to a significant decrease in the abundance of several other common bacteria, mainly including Christensenellaceae, *Christensenellaceae R7 group*, Oscillospiraceae, *UCG 002*, *UCG 005*, Rikenellaceae, and *Rikenellaceae RC9 gut group*. Notably, the deworming treatment in this study led to a significant decrease in the relative abundance of Clostridia in the gut of *R. brelichi*, which is consistent with the findings of Lee et al. (2019), Peachey et al. (2019), and Appiah-Twum et al. (2023). These studies reported that Clostridia can enhance the host’s gastrointestinal defense ability against parasitic infections through pathways such as competitive inhibition, immune cell priming, and regulation of immune tolerance. These comparative investigations into the characteristics of the gut microbiota before and after deworming, as well as in cases of successful and failed deworming, suggested that the maintenance of a high abundance of Clostridia after deworming can serve as an indicator of deworming failure. The significant decrease in the relative abundance of Clostridia and the significant reduction in EPG after deworming indicate that the deworming treatment in the present study was effective. Moreover, based on the aforementioned changes in the bacterial communities, it is believed that the significant decrease in Clostridia after deworming treatment in this study is attributable to the marked decline in the relative abundances of the family Christensenellaceae and its genus *Christensenellaceae R7 group*, the family Oscillospiraceae and its genera *UCG 002* and *UCG 005*. This finding will prompt us to verify the interaction mechanism between these bacterial communities and parasitic infections in subsequent research.

Analysis of the correlation network among bacterial communities uncovered extensive correlations between the biomarkers. A common pattern emerged: biomarkers within the same group generally showed positive correlations. For example, within the bacterial communities enriched in the intestines of *R. brelichi* before deworming, the *Christensenellaceae R7 group* demonstrated a positive correlation with *UCG 002*, and *UCG 005* was positively correlated with the *Rikenellaceae RC9 gut group*. Simultaneously, negative correlations frequently occurred between inter-group biomarkers. For instance, the *Christensenellaceae R7 group* was negatively correlated with *Prevotella 9*, and *UCG 005* was negatively correlated with both *Bacteroides* and *unclassified Muribaculaceae*. Notably, *unclassified Muribaculaceae* had the most extensive associations. It was positively correlated with *Bacteroides* yet negatively correlated with the *Rikenellaceae RC9 gut group* and *UCG 005*. The bacteria belonging to the family *Muribaculaceae* play a crucial role in the degradation of complex polysaccharides. Moreover, it has been reported that *unclassified Muribaculaceae* is crucial for alleviating inflammation, improving intestinal structure, and resisting coccidia infection (Yu et al., 2021; Liu et al., 2025). Meanwhile, besides degrading plant polysaccharides, *Bacteroides* has the ability to transport polysaccharide A to immune cells, thereby facilitating the release of the anti-inflammatory factor IL-10 and fulfilling an immunoprotective function (Meng et al., 2024). Research has indicated that cellulose can modulate the release of inflammatory factors by altering the abundances of bacterial communities such as *unclassified Muribaculaceae*, *Bacteroides*, and *Faecalibaculum*, thus exerting an immunomodulatory effect (Cao et al., 2024). In the present study, the significantly up-regulated *unclassified Muribaculaceae* following deworming treatment was positively correlated with the up-regulated *Bacteroides* and also positively correlated with *Faecalibaculum*. A specific study has revealed that the relative abundance of *Faecalibaculum rodentium* significantly decreases after mice are infected with *Trypanosoma cruzi*. Metagenomics analysis indicates that *Faecalibaculum rodentium* is closely associated with amino acid metabolism, nucleotide metabolism, and the biosynthetic pathway to cobalamin (Castañeda et al., 2023). Moreover, other research has shown that *Faecalibaculum*, a SCFA producer, is closely linked to both the anti-inflammatory cytokine IL-10 and the pro-inflammatory cytokine macrophage migration inhibitory factor (Xie et al., 2022; Cao et al., 2024). Notably, in the current study, the bacterial communities whose abundances are altered following deworming treatment exhibit extremely strong correlations with *Faecalibaculum*. These correlationships further suggests that these communities possess a certain degree of synergistic effect in protecting the host from infection.

Metabolomics represents a powerful tool for identifying metabolites either upregulated or downregulated by differential microbiota, thereby enabling a more in-depth exploration of the mechanism through which deworming treatment impacts host intestinal homeostasis. Through metabolomic analysis, we observed that the relative abundances of 382 out of 1,865 metabolites in the fecal samples changed significantly before and after deworming treatment. Lipids and lipid-like molecules exhibited the most prominent changes. These mainly included Prenol lipids, Glycerophospholipids, Fatty Acyls, Steroids and steroid derivatives, and Glycerolipids. They are involved in a wide range of biological processes, such as biological membranes stabilization, material transport, signal transduction, energy metabolism, and antioxidant activity (Blázquez et al., 2025; Ge et al., 2025; Jiang et al., 2025). In the gut of patients infected with *Schistosoma japonicum*, Lipids and lipid-like molecules were the class of metabolites with the most pronounced variations (Zhou et al., 2023). KEGG enrichment analysis indicated that the differential metabolites were mainly engaged in energy, amino acid, lipid, and purine metabolic processes. These processes encompassed ABC transporters, Phenylalanine metabolism, Purine metabolism, Ubiquinone and other terpenoid-quinone biosynthesis, Glycerophospholipid metabolism, Pyrimidine metabolism, Tryptophan metabolism, alpha-Linolenic acid metabolism, Bile secretion, Biosynthesis of amino acids, and Biosynthesis of unsaturated fatty acids, etc. The Pathways enriched in this study were comparable to those previously reported. For instance, after infecting mice with *Schistosoma japonicum*, significant disruptions were also found in Purine metabolism, Glycerophospholipid metabolism, and Pyrimidine metabolism (Hu et al., 2020). Treatment with praziquantel not only significantly inhibited the granulomatous reaction induced by *Schistosoma japonicum* eggs in the liver of mice but also substantially reduced the elevated abundance of pyrimidine metabolism during the chronic infection stage (Xue et al., 2024). At the cellular level, after EPC cells were *in vitro* infected with *Saprolegnia parasitica*, the differential metabolites were mainly enriched in ABC transporters, Glycerophospholipid metabolism, and Purine metabolism (Wang et al., 2025). Infection with *Echinococcus granulosus* could lead to significant alterations in the metabolism of various amino acids, such as phenylalanine and glycine (Bai et al., 2023). Ubiquinone and other terpenoid-quinone biosynthesis are widely present in animals, parasites, and bacteria. They play roles in antioxidation, electron transfer, energy production, cell signal transduction, as well as the synthesis of other biomolecules (Lai et al., 2023; Li et al., 2024). Disorders in Ubiquinone and other terpenoid-quinone biosynthesis can contribute to the onset and progression of various diseases, such as chronic kidney disease and thrombosis (Wang et al., 2022; Wang et al., 2023). In patients with severe *Ascaris lumbricoides* infection, the metabolites involved in alpha-linolenic acid metabolism in the gut increased significantly (Klomkliew et al., 2022). These metabolic pathways, which were significantly affected by parasite infections, also showed notable changes after the host underwent deworming treatment. It is speculated that the deworming treatment attempts to rectify the metabolic abnormalities in the host induced by parasite infections.

From the correlation analysis results, we discerned multiple relationships between the differential core microbiota and metabolites. The *Christensenellaceae R7 group*, whose relative abundance decreased after deworming treatment, demonstrated an important regulatory role in host metabolism. For instance, the *Christensenellaceae R7 group* was positively correlated with Retinyl beta-glucuronide. This compound, a reserve form of vitamin A, plays a pivotal role in the mucosal barriers and immune function of animals (Sánchez-Mendoza et al., 2024). Conversely, the *Christensenellaceae R7 group* showed a strong negative correlation with multiple metabolites, including CDP-Choline, 2-(Formamido)-N1-(5-phospho-D-ribosyl) acetamidine, Kaempferol, 5,7-Dihydroxy-3-methoxy-4′-prenyloxyflavone, carboxin, Purpurin, and Rhein. Numerous studies have reported that CDP-Choline is an important substance for the successful infection of the host by *Entamoeba histolytica* and *Plasmodium falciparum* (Chang et al., 2023; Teh et al., 2023). Kaempferol and 5,7-Dihydroxy-3-methoxy-4′-prenyloxyflavone belong to flavonoids, which possess a wide array of biological functions. Notably, Kaempferol has been reported to have anti-parasitic effects against various parasites such as *Schistosoma mansoni*, *Encephalitozoon intestinalis*, and *Naegleria fowleri* (Lê et al., 2023; Albuquerque et al., 2024; Gülpinar et al., 2025). Purpurin and Rhein are natural anthraquinones derived from plants. They have also been proven to possess high inhibitory activity against parasites of the genus *Trichomonas* (Friedman et al., 2020). Moreover, Rhein exhibited anti-inflammatory effects by increasing the abundance of *Lactobacillus* in the host gut and reducing intestinal permeability (Wu et al., 2020; Cho et al., 2024; Xie et al., 2024). In summary, the down-regulation of the *Christensenellaceae R7 group* induced by deworming treatment can, to a certain extent, enhance the ability of *R. brelichi* to resist parasite infections. However, during the deworming phase, additional supplementation with vitamin A and attention to inhibiting the production of CDP-Choline will help mitigate the side effects of the deworming treatment. The multiple associations between the microbiota and metabolites are also manifested in several ways. Metabolites with anti-parasitic activity such as Kaempferol, 5,7-Dihydroxy-3-methoxy-4′-prenyloxyflavone, Purpurin, and Rhein were negatively correlated with the *Christensenellaceae R7 group*. Additionally, they were positively correlated with *Bacteroides* and *unclassified Muribaculaceae*, whose relative abundances increased significantly after deworming treatment. This indicates that the changes in these metabolites are regulated by the combined actions of multiple bacterial communities. In addition, the relative abundances of *UCG 002*, *UCG 005*, *Rikenellaceae RC9 gut group*, and *uncultured rumen bacterium*, which decreased after deworming treatment, were negatively correlated with Palmitaldehyde and positively correlated with Gibberellin A14. Palmitaldehyde is a volatile long-chain fatty aldehyde associated with Fatty acid degradation (Wang L. et al., 2024). Gibberellin A14 has been reported to be involved in the production of antibiotic resistance genes (ARG) in the cecal microbiota of laying hens (Xing et al., 2021). Based on these two set of relationships, it is speculated that the decrease in the relative abundances of these four bacterial communities after deworming treatment promotes the degradation of fatty acids and, to some extent, reduces the formation or expression of antibiotic resistance genes in the gut microbiota of *R. brelichi*.

The present study has two main limitations. First, the study duration was relatively short. In future research, longitudinal experiments will be incorporated to monitor the long-term impacts of albendazole deworming on the gut microbiota and its metabolic functions. Second, in this study, only the EPG values before and after deworming were calculated, and no molecular identification of the parasites was carried out. As a result, this research is mainly an analysis of the characteristics of the gut microbiota and its metabolism before and after deworming. It remains challenging to determine whether the observed changes are a direct consequence of albendazole or are due to alterations in the parasitic status. Considering that *R. brelichi* is an endangered wild protected species, in subsequent experiments, the relationships between the differential microbiota and metabolites following deworming treatment will be validated using experimental animals. Additionally, experimental animals will be inoculated with the dominant parasites found in *R. brelichi* to explore the influence of specific parasites on the gut microbiota and its metabolic functions.

## Conclusion

In this study, multi-omics analysis methods were employed to explore the characteristics of changes in gut microbiota and metabolites within captive *R. brelichi* both prior to and following deworming. Some potential correlations were discovered among the differential microbiota and metabolites. Specifically, the family Christensenellaceae along with its genus *Christensenellaceae R7 group*, as well as the family Oscillospiraceae along with its genera *UCG 002* and *UCG 005*, all of which are classified under Clostridia, hold promise as potential indicators for assessing the efficacy of deworming regimens. Moreover, *Bacteroides* and *unclassified Muribaculaceae* can be used as potential probiotics to improve the deworming effect. In addition, to reduce the side effects of deworming, attention should be paid to supplementing vitamin A and inhibiting the production of CDP-Choline.

## Data Availability

The datasets presented in this study can be found in online repositories. The names of the repository/repositories and accession number(s) can be found below: https://www.ncbi.nlm.nih.gov/, PRJNA1225780.

## Footnotes

1
www.iucnredlist.org/
