# Supplementary material for: Multi-omics insights into the response of the gut microbiota and metabolites to albendazole deworming in captive Rhinopithecus brelichi
Source: Front Microbiol. 2025 Apr 23;16:1581483. doi: 10.3389/fmicb.2025.1581483 (PMC12058082; doi:10.3389/fmicb.2025.1581483)
Supplement: Supplementary file 1 [file Image_1.pdf]

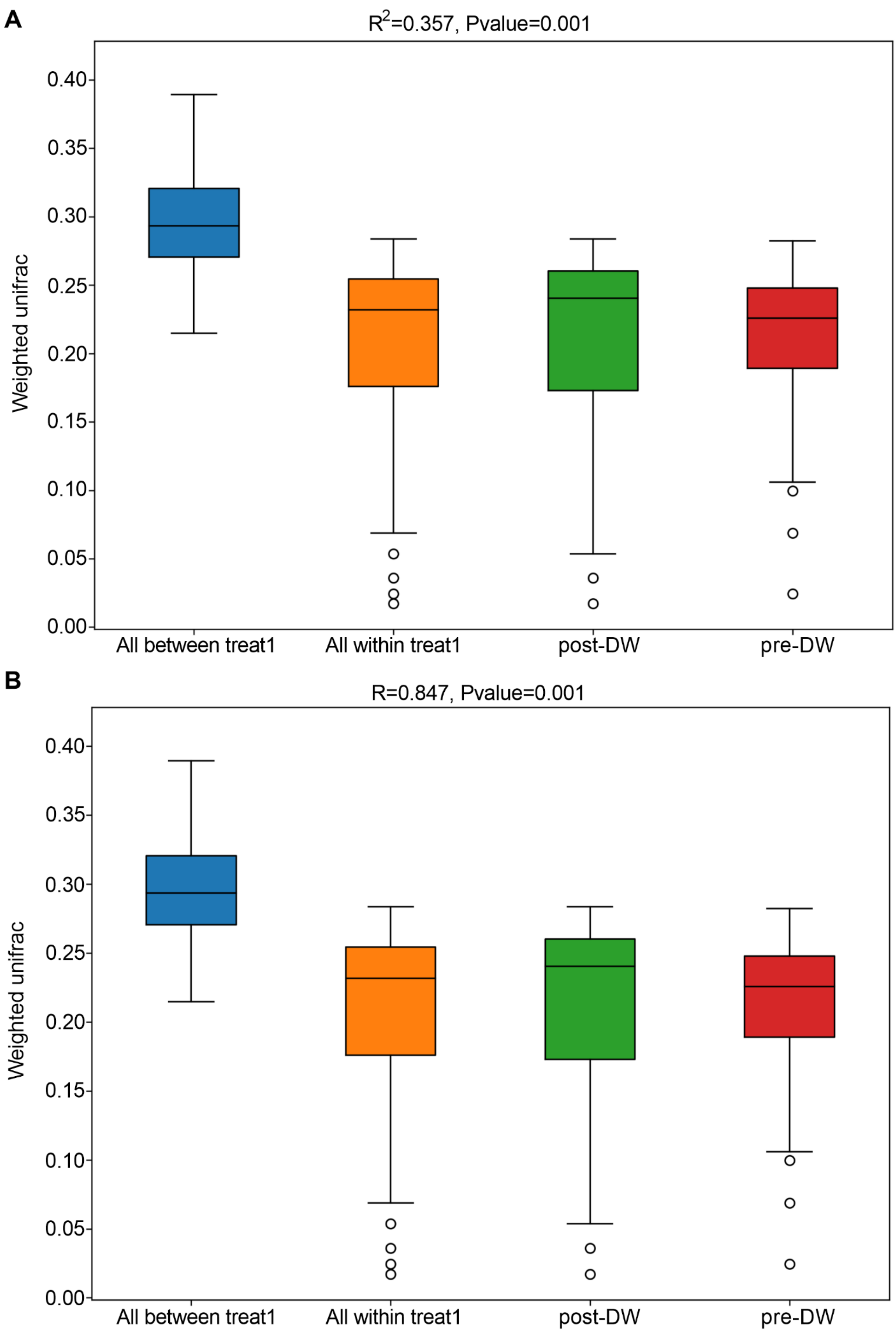

Supplementary Figure S1. Analysis of significant differences in beta diversity of samples between groups. (A) Permanova analysis. The R^2 value represents the degree of explanation for differences among samples from different groups. A larger R^2 value implies that the grouping has a higher

explanatory power for the observed differences. A *P* value of less than 0.05 indicates a high level of reliability for the test. (B) Anosim analysis. The closer the *R* value is to 1, the higher the difference between groups is than that within groups, *P* value less than 0.05 indicates high reliability of the test. Distance matrix: weighted unifrac. DW: fecal samples before deworming treatment; post-DW: fecal samples after deworming treatment.
