# Supplementary material for: Multi-omics insights into the response of the gut microbiota and metabolites to albendazole deworming in captive Rhinopithecus brelichi
Source: Front Microbiol. 2025 Apr 23;16:1581483. doi: 10.3389/fmicb.2025.1581483 (PMC12058082; doi:10.3389/fmicb.2025.1581483)
Supplement: Supplementary file 2 [file Table_1.docx]

Supplementary Table S1 Data processing results

| Sample  ID | Raw  Reads | Clean  Reads | Denoised Reads | Merged Reads | Non-chimeric Reads |
| --- | --- | --- | --- | --- | --- |
| pre-DW1 | 79,871 | 79,655 | 78,052 | 75,401 | 73,274 |
| pre-DW2 | 80,425 | 80,200 | 77,934 | 69,182 | 64,972 |
| pre-DW3 | 79,798 | 79,569 | 78,101 | 73,664 | 71,250 |
| pre-DW4 | 80,247 | 80,020 | 77,969 | 72,154 | 69,732 |
| pre-DW5 | 80,307 | 80,059 | 78,381 | 73,481 | 71,146 |
| pre-DW6 | 80,225 | 79,993 | 77,430 | 67,816 | 61,944 |
| pre-DW7 | 80,136 | 79,860 | 76,436 | 61,484 | 54,006 |
| pre-DW8 | 80,037 | 79,816 | 78,319 | 73,463 | 70,124 |
| pre-DW9 | 79,912 | 79,708 | 78,237 | 74,065 | 71,199 |
| pre-DW10 | 80,216 | 80,003 | 78,164 | 73,934 | 70,438 |
| post-DW1 | 80,062 | 79,835 | 78,039 | 72,304 | 69,084 |
| post-DW2 | 82,979 | 82,712 | 80,011 | 70,472 | 62,291 |
| post-DW3 | 80,134 | 79,902 | 78,822 | 76,316 | 74,359 |
| post-DW4 | 79,853 | 79,637 | 78,129 | 74,338 | 72,401 |
| post-DW5 | 80,198 | 79,979 | 78,218 | 72,370 | 70,062 |
| post-DW6 | 80,383 | 80,151 | 78,703 | 74,175 | 72,067 |
| post-DW7 | 79,925 | 79,703 | 78,200 | 73,596 | 71,541 |
| post-DW8 | 79,884 | 79,669 | 77,532 | 69,919 | 66,699 |
| post-DW9 | 79,980 | 79,786 | 77,954 | 71,044 | 68,228 |
| post-DW10 | 79,648 | 79,390 | 77,601 | 73,541 | 71,714 |
