# Supplementary material for: Multi-omics insights into the response of the gut microbiota and metabolites to albendazole deworming in captive Rhinopithecus brelichi
Source: Front Microbiol. 2025 Apr 23;16:1581483. doi: 10.3389/fmicb.2025.1581483 (PMC12058082; doi:10.3389/fmicb.2025.1581483)
Supplement: Supplementary file 3 [file Table_2.docx]

Supplementary Table S2 Differences in relative abundance of the identified phyla between the pre- and post-deworming groups (Wilcoxon rank-sum test, and *P*-values were corrected using FDR).

| Phylum | pre-DW  (%) | post-DW  (%) | pre-DW vs  post-DW (*P*) |
| --- | --- | --- | --- |
| Firmicutes | 65.05 | 52.66 | 0.004 |
| Bacteroidota | 19.12 | 31.52 | 0.002 |
| Spirochaetota | 4.20 | 5.82 | 0.587 |
| Verrucomicrobiota | 4.30 | 2.28 | 0.889 |
| Proteobacteria | 0.91 | 2.27 | 0.008 |
| Cyanobacteria | 0.25 | 1.56 | 0.004 |
| Fibrobacterota | 0.29 | 1.30 | 0.008 |
| Desulfobacterota | 4.79 | 0.28 | 0.002 |
| Elusimicrobiota | 0.07 | 0.20 | 0.527 |
| Actinobacteriota | 0.13 | 0.14 | 0.880 |
| Campylobacterota | 0.14 | 0.06 | 0.009 |
| Fusobacteriota | 0.01 | 0.00 | 0.371 |
| unclassified_Bacteria | 0.74 | 1.91 | 0.051 |
