# Supplementary material for: Multi-omics insights into the response of the gut microbiota and metabolites to albendazole deworming in captive Rhinopithecus brelichi
Source: Front Microbiol. 2025 Apr 23;16:1581483. doi: 10.3389/fmicb.2025.1581483 (PMC12058082; doi:10.3389/fmicb.2025.1581483)
Supplement: Supplementary file 4 [file Table_3.docx]

Supplementary Table S3 Differences in relative abundance of the identified genera between the pre- and post-deworming groups (Wilcoxon rank-sum test, and *P*-values were corrected using FDR).

| Genus | pre-DW  (%) | post-DW  (%) | pre-DW vs  post-DW (*P*) |
| --- | --- | --- | --- |
| *unclassified Muribaculaceae* | 2.43 | 11.37 | 0.008 |
| *UCG 005* | 10.91 | 5.41 | 0.008 |
| *Bacteroides* | 1.09 | 4.59 | 0.008 |
| *uncultured rumen bacterium* | 9.67 | 4.06 | 0.008 |
| *Alistipes* | 0.48 | 2.27 | 0.012 |
| *Rikenellaceae RC9 gut group* | 7.49 | 1.60 | 0.009 |
| *Faecalibacterium* | 0.34 | 1.46 | 0.024 |
| *Phascolarctobacterium* | 3.32 | 1.43 | 0.049 |
| *Fibrobacter* | 0.29 | 1.30 | 0.028 |
| *unclassified Rhodospirillales* | 0.39 | 1.30 | 0.049 |
| *UCG 009* | 1.55 | 0.61 | 0.025 |
| *Parasutterella* | 0.14 | 0.52 | 0.049 |
| *unclassified Verrucomicrobiae* | 0.07 | 0.52 | 0.027 |
| *unclassified Gastranaerophilales* | 0.02 | 0.43 | 0.007 |
| *Lachnospiraceae UCG 010* | 0.03 | 0.31 | 0.008 |
| *Candidatus Soleaferrea* | 0.48 | 0.23 | 0.034 |
| *unclassified RF39* | 0.51 | 0.16 | 0.045 |
| *unclassified Oscillospirales* | 0.38 | 0.15 | 0.041 |
| *[Eubacterium] eligens group* | 0.00 | 0.13 | 0.008 |
| *Tyzzerella* | 0.00 | 0.12 | 0.009 |
| *unclassified Erysipelotrichaceae* | 0.00 | 0.08 | 0.012 |
| *unclassified Veillonellales Selenomonadales* | 0.00 | 0.07 | 0.006 |
| *uncultured Lachnospiraceae bacterium* | 0.00 | 0.07 | 0.044 |
| *uncultured Firmicutes bacterium* | 0.39 | 0.05 | 0.048 |
| *uncultured Clostridium sp.* | 0.14 | 0.01 | 0.007 |
| *unclassified Izemoplasmatales* | 0.17 | 0.00 | 0.047 |
| *unclassified [Eubacterium] coprostanoligenes group* | 5.53 | 7.21 | 0.728 |
| *unclassified Lachnospiraceae* | 4.32 | 7.17 | 0.173 |
| *Treponema* | 4.20 | 5.82 | 0.647 |
| *Christensenellaceae R 7 group* | 6.48 | 4.12 | 0.122 |
| *Prevotella 9* | 0.01 | 3.81 | 0.086 |
| *UCG 002* | 5.23 | 2.80 | 0.117 |
| *unclassified UCG 010* | 2.70 | 2.29 | 0.608 |
| *Ruminococcus* | 2.93 | 2.20 | 0.653 |
| *unclassified Bacteroidales* | 1.12 | 1.96 | 0.483 |
| *unclassified Bacteria* | 0.74 | 1.91 | 0.114 |
| *NK4A214 group* | 3.22 | 1.85 | 0.306 |
| *Parabacteroides* | 1.37 | 1.84 | 0.929 |
| *unclassified Prevotellaceae* | 2.03 | 1.50 | 0.642 |
| *Monoglobus* | 1.07 | 1.49 | 0.965 |
| *unclassified Clostridia UCG 014* | 2.46 | 1.38 | 0.357 |
| *unclassified Ruminococcaceae* | 0.84 | 1.27 | 0.276 |
| *Prevotellaceae UCG 001* | 0.41 | 1.21 | 0.053 |
| *Lachnospira* | 0.17 | 0.97 | 0.056 |
| *Prevotellaceae UCG 003* | 0.22 | 0.63 | 0.754 |
| *Anaerovibrio* | 0.37 | 0.61 | 0.377 |
| *[Eubacterium] siraeum group* | 0.31 | 0.57 | 0.622 |
| *Family XIII AD3011 group* | 0.43 | 0.57 | 0.919 |
| *Roseburia* | 0.34 | 0.54 | 0.713 |
| *unclassified Clostridia* | 0.33 | 0.50 | 0.051 |
| *Butyrivibrio* | 0.17 | 0.49 | 0.609 |
| *Oscillibacter* | 0.62 | 0.44 | 0.109 |
| *[Eubacterium] ruminantium group* | 0.57 | 0.34 | 0.314 |
| *unclassified Oscillospiraceae* | 0.45 | 0.33 | 0.301 |
| *CAG 352* | 0.05 | 0.33 | 0.232 |
| *Marvinbryantia* | 0.26 | 0.32 | 0.575 |
| *unclassified Clostridia vadinBB60 group* | 1.05 | 0.30 | 0.912 |
| *Prevotella 7* | 0.55 | 0.29 | 0.258 |
| *Desulfovibrio* | 0.17 | 0.28 | 0.642 |
| *Blautia* | 0.16 | 0.21 | 0.531 |
| *unclassified Christensenellaceae* | 0.70 | 0.21 | 0.176 |
| *Elusimicrobium* | 0.07 | 0.20 | 0.602 |
| *Alloprevotella* | 0.00 | 0.20 | 0.058 |
| *Coprococcus* | 0.15 | 0.17 | 0.286 |
| *[Eubacterium] nodatum group* | 0.09 | 0.16 | 0.441 |
| *Sutterella* | 0.06 | 0.15 | 0.120 |
| *Lachnospiraceae ND3007 group* | 0.11 | 0.14 | 0.550 |
| *[Eubacterium] ventriosum group* | 0.16 | 0.14 | 0.372 |
| *unclassified Hungateiclostridiaceae* | 0.06 | 0.13 | 0.507 |
| *unclassified p 2534 18B5 gut group* | 0.29 | 0.13 | 0.297 |
| *unclassified Peptococcaceae* | 0.10 | 0.12 | 0.613 |
| *UCG 004* | 1.64 | 0.11 | 0.080 |
| *uncultured prokaryote* | 0.21 | 0.11 | 0.563 |
| *Lachnospiraceae UCG 003* | 0.05 | 0.11 | 0.464 |
| *UCG 003* | 0.06 | 0.10 | 0.443 |
| *unclassified Cyanobacteriales* | 0.01 | 0.10 | 0.460 |
| *[Eubacterium] xylanophilum group* | 0.08 | 0.09 | 0.597 |
| *Dorea* | 0.09 | 0.09 | 0.794 |
| *Lachnospiraceae NK4A136 group* | 0.02 | 0.08 | 0.228 |
| *Incertae Sedis* | 0.04 | 0.08 | 0.201 |
| *Parvibacter* | 0.07 | 0.07 | 0.774 |
| *Butyricimonas* | 0.01 | 0.06 | 0.060 |
| *[Ruminococcus] gauvreauii group* | 0.00 | 0.06 | 0.180 |
| *Olsenella* | 0.03 | 0.06 | 0.630 |
| *Akkermansia* | 0.11 | 0.05 | 0.734 |
| *unclassified Bacteroidales RF16 group* | 1.45 | 0.05 | 0.051 |
| *Negativibacillus* | 0.03 | 0.05 | 0.780 |
| *uncultured Clostridiales bacterium* | 0.10 | 0.05 | 0.923 |
| *Limosilactobacillus* | 0.01 | 0.05 | 0.274 |
| *horsej a03* | 0.03 | 0.05 | 0.917 |
| *uncultured Erysipelotrichaceae bacterium* | 0.10 | 0.05 | 0.905 |
| *Colidextribacter* | 0.07 | 0.05 | 0.834 |
| *unclassified Peptostreptococcaceae* | 0.04 | 0.04 | 0.976 |
| *GCA 900066575* | 0.00 | 0.04 | 0.051 |
| *unclassified Alphaproteobacteria* | 0.17 | 0.04 | 0.281 |
| *Fournierella* | 0.03 | 0.03 | 0.979 |
| *Family XIII UCG 001* | 0.04 | 0.03 | 0.924 |
| *Campylobacter* | 0.06 | 0.03 | 0.161 |
| *Intestinimonas* | 0.00 | 0.03 | 0.636 |
| *unclassified Anaerovoracaceae* | 0.14 | 0.03 | 0.305 |
| *Oxalobacter* | 0.02 | 0.03 | 0.525 |
| *Rheinheimera* | 0.00 | 0.03 | 0.297 |
| *Helicobacter* | 0.08 | 0.03 | 0.293 |
| *Lachnospiraceae NC2004 group* | 0.00 | 0.03 | 0.470 |
| *Lachnospiraceae UCG 001* | 0.05 | 0.03 | 0.972 |
| *Pelomonas* | 0.00 | 0.03 | 0.301 |
| *Azospirillum sp. 47 25* | 0.00 | 0.03 | 0.092 |
| *Oribacterium* | 0.03 | 0.02 | 0.936 |
| *Anaerovorax* | 0.00 | 0.02 | 0.053 |
| *Dielma* | 0.00 | 0.02 | 0.583 |
| *Peptococcus* | 0.02 | 0.02 | 0.419 |
| *Hydrogenophaga* | 0.00 | 0.02 | 0.311 |
| *unclassified vadinBE97* | 0.03 | 0.02 | 0.656 |
| *unclassified Puniceicoccaceae* | 0.02 | 0.01 | 0.662 |
| *Sanguibacteroides* | 0.00 | 0.01 | 0.125 |
| *Lachnospiraceae NK4B4 group* | 0.06 | 0.01 | 0.117 |
| *Paludicola* | 0.00 | 0.01 | 0.459 |
| *Erysipelotrichaceae UCG 003* | 0.00 | 0.01 | 0.243 |
| *Frisingicoccus* | 0.01 | 0.01 | 1.000 |
| *Lactobacillus* | 0.04 | 0.01 | 0.788 |
| *Subdoligranulum* | 0.00 | 0.01 | 0.292 |
| *Escherichia Shigella* | 0.03 | 0.01 | 0.931 |
| *Dechloromonas* | 0.01 | 0.01 | 0.990 |
| *Acidovorax* | 0.01 | 0.01 | 0.740 |
| *Coprobacillus* | 0.00 | 0.01 | 0.800 |
| *unclassified Leptolyngbyaceae* | 0.00 | 0.01 | 0.983 |
| *Enterorhabdus* | 0.01 | 0.00 | 0.667 |
| *Novosphingobium* | 0.01 | 0.00 | 0.943 |
| *[Eubacterium] brachy group* | 0.01 | 0.00 | 0.828 |
| *Actinomyces* | 0.01 | 0.00 | 0.494 |
| *Methylotenera* | 0.01 | 0.00 | 0.569 |
| *unclassified Eggerthellaceae* | 0.02 | 0.00 | 0.269 |
| *Clostridium sensu stricto 1* | 0.04 | 0.00 | 0.589 |
| *unclassified Butyricicoccaceae* | 0.02 | 0.00 | 0.158 |
| *Anaeroplasma* | 0.19 | 0.00 | 0.056 |
| *Anaerosporobacter* | 0.02 | 0.00 | 0.814 |
| *Anaerostipes* | 0.17 | 0.00 | 0.488 |
| *Candidatus Stoquefichus* | 0.02 | 0.00 | 0.649 |
| *Caproiciproducens* | 0.02 | 0.00 | 0.482 |
| *Cetobacterium* | 0.01 | 0.00 | 0.476 |
| *Coxiella* | 0.03 | 0.00 | 0.808 |
| *Lachnospiraceae AC2044 group* | 0.07 | 0.00 | 0.184 |
| *Prevotella* | 0.02 | 0.00 | 0.625 |
| *Prevotellaceae NK3B31 group* | 0.02 | 0.00 | 0.453 |
| *T2WK15B57* | 0.24 | 0.00 | 0.448 |
| *[Eubacterium] hallii group* | 0.02 | 0.00 | 0.619 |
| *dgA 11 gut group* | 0.14 | 0.00 | 0.288 |
| *uncultured Alphaproteobacteria bacterium* | 0.03 | 0.00 | 0.438 |
| *uncultured Clostridia bacterium* | 0.08 | 0.00 | 0.602 |
| *uncultured Ruminococcaceae bacterium* | 0.12 | 0.00 | 0.597 |
