# Supplementary material for: Multi-omics insights into the response of the gut microbiota and metabolites to albendazole deworming in captive Rhinopithecus brelichi
Source: Front Microbiol. 2025 Apr 23;16:1581483. doi: 10.3389/fmicb.2025.1581483 (PMC12058082; doi:10.3389/fmicb.2025.1581483)
Supplement: Supplementary file 5 [file Table_4.docx]

Supplementary Table S4 Annotation of metabolites detected by UHPLC-Q-TOF-MS based on HMDB 4.0 database

| SuperClass | HMDB taxonomy | Metabolites numbers |
| --- | --- | --- |
| Organoheterocyclic compounds | Azoles | 46 |
| Organoheterocyclic compounds | Benzazepines | 62 |
| Organoheterocyclic compounds | Benzimidazoles | 20 |
| Organoheterocyclic compounds | Benzodiazepines | 125 |
| Organoheterocyclic compounds | Benzodioxoles | 75 |
| Organoheterocyclic compounds | Benzofurans | 5 |
| Organoheterocyclic compounds | Benzopyrans | 67 |
| Organoheterocyclic compounds | Benzoxazines | 134 |
| Organoheterocyclic compounds | Biotin and derivatives | 56 |
| Organoheterocyclic compounds | Coumarans | 73 |
| Organoheterocyclic compounds | Diazinanes | 68 |
| Organoheterocyclic compounds | Diazines | 42 |
| Organoheterocyclic compounds | Dihydrofurans | 76 |
| Organoheterocyclic compounds | Dioxanes | 52 |
| Organoheterocyclic compounds | Dioxolanes | 74 |
| Organoheterocyclic compounds | Epoxides | 54 |
| Organoheterocyclic compounds | Furans | 3 |
| Organoheterocyclic compounds | Furofurans | 41 |
| Organoheterocyclic compounds | Heteroaromatic compounds | 112 |
| Organoheterocyclic compounds | Imidazopyrimidines | 61 |
| Organoheterocyclic compounds | Indoles and derivatives | 41 |
| Organoheterocyclic compounds | Isobenzofurans | 72 |
| Organoheterocyclic compounds | Isocoumarans | 77 |
| Organoheterocyclic compounds | Isoquinolines and derivatives | 63 |
| Organoheterocyclic compounds | Lactams | 53 |
| Organoheterocyclic compounds | Lactones | 50 |
| Organoheterocyclic compounds | Naphthofurans | 69 |
| Organoheterocyclic compounds | Oxazinanes | 76 |
| Organoheterocyclic compounds | Oxepanes | 92 |
| Organoheterocyclic compounds | Piperidines | 48 |
| Organoheterocyclic compounds | Pteridines and derivatives | 94 |
| Organoheterocyclic compounds | Pyrans | 79 |
| Organoheterocyclic compounds | Pyridines and derivatives | 60 |
| Organoheterocyclic compounds | Pyridopyrimidines | 49 |
| Organoheterocyclic compounds | Pyrrolidines | 28 |
| Organoheterocyclic compounds | Pyrrolopyrimidines | 46 |
| Organoheterocyclic compounds | Quinolines and derivatives | 96 |
| Organoheterocyclic compounds | Tetrahydrofurans | 22 |
| Organoheterocyclic compounds | Tetrahydroisoquinolines | 49 |
| Organoheterocyclic compounds | Tetrapyrroles and derivatives | 66 |
| Organoheterocyclic compounds | Triazines | 55 |
| Lipids and lipid-like molecules | Endocannabinoids | 232 |
| Lipids and lipid-like molecules | Fatty Acyls | 124 |
| Lipids and lipid-like molecules | Glycerolipids | 354 |
| Lipids and lipid-like molecules | Glycerophospholipids | 292 |
| Lipids and lipid-like molecules | Prenol lipids | 287 |
| Lipids and lipid-like molecules | Sphingolipids | 358 |
| Lipids and lipid-like molecules | Steroids and steroid derivatives | 294 |
| Benzenoids | Anthracenes | 109 |
| Benzenoids | Benzene and substituted derivatives | 75 |
| Benzenoids | Dibenzocycloheptenes | 67 |
| Benzenoids | Indanes | 66 |
| Benzenoids | Naphthalenes | 119 |
| Benzenoids | Phenanthrenes and derivatives | 71 |
| Benzenoids | Phenol esters | 208 |
| Benzenoids | Phenols | 99 |
| Benzenoids | Pyrenes | 25 |
| Benzenoids | Tetralins | 82 |
| Organic acids and derivatives | Carboximidic acids and derivatives | 69 |
| Organic acids and derivatives | Carboxylic acids and derivatives | 117 |
| Organic acids and derivatives | Hydroxy acids and derivatives | 59 |
| Organic acids and derivatives | Keto acids and derivatives | 125 |
| Organic acids and derivatives | Organic carbonic acids and derivatives | 131 |
| Organic acids and derivatives | Organic phosphonic acids and derivatives | 128 |
| Organic acids and derivatives | Organic phosphoric acids and derivatives | 64 |
| Organic acids and derivatives | Organic sulfuric acids and derivatives | 131 |
| Organic acids and derivatives | Peptidomimetics | 137 |
| Phenylpropanoids and polyketides | 2-arylbenzofuran flavonoids | 55 |
| Phenylpropanoids and polyketides | 3,4-dihydrocoumarins | 36 |
| Phenylpropanoids and polyketides | Cinnamaldehydes | 42 |
| Phenylpropanoids and polyketides | Cinnamic acids and derivatives | 25 |
| Phenylpropanoids and polyketides | Cinnamyl alcohols | 23 |
| Phenylpropanoids and polyketides | Coumarins and derivatives | 8 |
| Phenylpropanoids and polyketides | Diarylheptanoids | 32 |
| Phenylpropanoids and polyketides | Flavonoids | 43 |
| Phenylpropanoids and polyketides | hydes | 37 |
| Phenylpropanoids and polyketides | Isoflavonoids | 50 |
| Phenylpropanoids and polyketides | Linear 1,3-diarylpropanoids | 12 |
| Phenylpropanoids and polyketides | Macrolides and analogues | 10 |
| Phenylpropanoids and polyketides | Phenylpropanoic acids | 34 |
| Phenylpropanoids and polyketides | Stilbenes | 60 |
| Nucleosides, nucleotides, and analogues | 5')-dinucleotides | 11 |
| Nucleosides, nucleotides, and analogues | 5'-deoxyribonucleosides | 25 |
| Nucleosides, nucleotides, and analogues | Flavin nucleotides | 22 |
| Nucleosides, nucleotides, and analogues | Glycinamide ribonucleotides | 21 |
| Nucleosides, nucleotides, and analogues | Nucleoside and nucleotide analogues | 14 |
| Nucleosides, nucleotides, and analogues | Purine nucleosides | 53 |
| Nucleosides, nucleotides, and analogues | Pyrimidine nucleosides | 64 |
| Nucleosides, nucleotides, and analogues | Ribonucleoside 3'-phosphates | 12 |
| Organic oxygen compounds | Organooxygen compounds | 95 |
| Organic nitrogen compounds | Organonitrogen compounds | 20 |
| Hydrocarbons | Unsaturated hydrocarbons | 11 |
| Alkaloids and derivatives | Harmala alkaloids | 3 |
| Alkaloids and derivatives | Morphinans | 2 |
| Alkaloids and derivatives | Tropane alkaloids | 4 |
| Alkaloids and derivatives | Yohimbine alkaloids | 1 |
| Organosulfur compounds | Thioethers | 3 |
| Organosulfur compounds | Thiols | 4 |
| Lignans, neolignans and related compounds | Furanoid lignans | 4 |
| Homogeneous non-metal compounds | Non-metal oxoanionic compounds | 2 |
| Hydrocarbon derivatives | Tropones | 2 |
